# Fine-Gradient Low-Molecular-Weight Hyaluronic Acid Supplementation Modulates Gut Microbial Profiles and SCFA Output in a Starch-Containing In Vitro Fecal Fermentation Model

**DOI:** 10.3390/microorganisms14051021

**Published:** 2026-04-30

**Authors:** Jie Dong, Tianyue Guan, Yuzheng Xue, Jinsong Shi, Zhenghong Xu, Yan Geng, Yilin Ren

**Affiliations:** 1 Key Laboratory of Carbohydrate Chemistry and Biotechnology, Ministry of Education, Affiliated Hospital of Jiangnan University, Jiangnan University, Wuxi 214125, China; 1162230130@stu.jiangnan.edu.cn (J.D.); 9862018034@jiangnan.edu.cn (Y.X.); 2 School of Life Sciences and Health Engineering, Jiangnan University, Wuxi 214122, China; 6191504018@stu.jiangnan.edu.cn (T.G.); shijs@jiangnan.edu.cn (J.S.); 3 Innovation Center for Advanced Brewing Science and Technology, Sichuan University, Chengdu 610065, China; zhenghxu@jiangnan.edu.cn

**Keywords:** microbiota-derived metabolites, hyaluronic acid, molecular weight, gut microbiota, in vitro fermentation, short-chain fatty acids, 16S rRNA gene sequencing

## Abstract

Hyaluronic acid (HA) is a glycosaminoglycan commonly administered orally, and its molecular weight (MW) influences its physicochemical behavior and potential interactions with the gut microbiota. However, MW-dependent effects on community assembly and fermentation-derived metabolites within the low-molecular-weight (LMW) range remain insufficiently resolved. In this study, five HA samples (6.9–35 kDa) were evaluated using an in vitro human fecal fermentation model. Microbial composition was profiled by 16S rRNA gene sequencing, and SCFAs were quantified by UPLC. Compared with the control under the same basal medium, HA supplementation was associated with shifts in community structure and higher alpha diversity. The 6.9 and 9.5 kDa groups were associated with significantly higher total SCFA concentrations, particularly butyrate, than the 13, 17, and 35 kDa groups under the same basal medium. Because soluble starch was present in the fermentation medium, these differences should be interpreted as modulation effects rather than direct evidence of HA-specific fermentation. 16S-based functional prediction suggested MW-dependent differences in predicted central carbohydrate metabolism potential, which were consistent with the observed SCFA patterns but should be interpreted as inferred functional potential rather than direct evidence of pathway activity. These findings indicate that HA molecular weight is associated with differential microbial and SCFA response patterns under the present in vitro conditions. Lower-MW HA within the tested range was associated with higher SCFA output, particularly butyrate, under a shared starch-containing basal medium, highlighting molecular weight as a potential formulation parameter in HA microbiota-centered applications.

## 1. Introduction

Short-chain fatty acids (SCFAs) are key microbiota-derived metabolites generated during colonic fermentation and are widely implicated in gut barrier and immune regulation [1]. Beyond live probiotics, microbiota-derived metabolites provide a mechanistic window into how microbial activity links diet to host physiology [2]. Among the diverse microbiota-derived metabolites, SCFAs—primarily acetate, propionate, and butyrate—are well-recognized microbial products that play pivotal roles in maintaining gut barrier integrity, regulating immune responses, and modulating host metabolic homeostasis [3,4].

Hyaluronic acid (HA, or hyaluronan) is a linear glycosaminoglycan composed of repeating disaccharide units and is widely used as an oral dietary supplement and functional food ingredient [5]. A critical determinant of its biological activity is its MW, which governs its physicochemical properties and biological interactions [6,7]. Importantly, due to its resistance to host digestive enzymes, orally administered HA can reach the colon largely intact, where it becomes accessible to gut microbial metabolism [7]. From a microbial perspective, HA may interact with gut microbial communities and influence fermentation-associated metabolic outputs under appropriate experimental conditions [8,9].

Notably, the MW dependency of HA gives rise to a well-documented paradox. High-molecular-weight (HMW) HA is generally associated with anti-inflammatory and tissue-protective effects, whereas LMW HA carries a risk of eliciting pro-inflammatory effects upon interaction with the host organism [10,11]. These observations have contributed to ongoing concerns regarding the suitability of LMW HA for dietary applications. However, such conclusions are mostly based on host-centric models, which primarily emphasize direct receptor-mediated inflammatory signaling but may not fully incorporate the potential contribution of downstream microbial fermentation processes [12,13].

The gut microbiota serves as a central metabolic interface that converts indigestible dietary components into bioactive microbiota-derived metabolites, including SCFAs, which exert profound effects on host physiology [8,14]. Indeed, previous studies have demonstrated that orally ingested HA can be utilized by gut microorganisms and fermented into SCFAs with anti-inflammatory potential [7]. Nevertheless, research on LMW HA has predominantly bypassed this critical microbial interface, focusing instead on its immediate host-level inflammatory pathways [15,16,17]. Neglecting the microbial contribution may therefore lead to an incomplete or even misleading interpretation of the biological effects of orally administered LMW HA [18].

Despite growing interest in HA–microbiota interactions, existing investigations into MW-dependent effects typically rely on comparisons across broad and heterogeneous MW ranges [5,9,19]. Such coarse classifications may obscure subtle yet biologically meaningful differences, particularly within the LMW spectrum that is most relevant for oral supplementation. Because polymer chain length may influence microbial interactions with polysaccharides, even fine-scale MW variations could be associated with distinct microbial community responses and SCFA profiles. Consequently, a systematic, fine-gradient analysis within the LMW range may help clarify whether MW is linked to differential microbiota-associated fermentation outcomes.

Therefore, in the present study, we adopted a microbiota–metabolite–oriented perspective, focusing not on direct host–receptor interactions but on microbiota-associated metabolic outputs under an in vitro fecal fermentation background. Rather than defining HA itself as a postbiotic or assuming HA-specific fermentation, we evaluated whether LMW HA supplementation is associated with differential microbial community configurations and fermentation-derived metabolite profiles, particularly SCFAs. To examine whether fine-scale molecular-weight differences within the LMW range are linked to distinct microbial and SCFA response patterns, we employed an in vitro human gut fermentation model to systematically analyze five LMW HAs spanning a fine MW gradient (6.9–35 kDa). By integrating 16S rRNA gene sequencing and quantitative SCFA profiling, we examined associations between HA MW, microbial community structure, and SCFA output under the present in vitro conditions. This study provides evidence that HA molecular weight is associated with distinct microbial and SCFA profiles under the present in vitro conditions, offering practical insights for the selection and formulation of HA as a functional food ingredient.

## 2. Materials and Methods

### 2.1. Preparation of Fecal Samples

Six healthy volunteers (3 women and 3 men), aged 20–30 years, were recruited. To ensure the stability of the gut microbiota, none of the participants had received antibiotics, probiotics, or prebiotics for at least 3 months prior to sample collection. The study was endorsed by the Medical Ethics Committee of Jiangnan University. Fresh fecal samples were collected from each participant using a sample collector. For each experimental group, nine anaerobic fermentation bottles were prepared. Six bottles were inoculated separately with fecal microbiota derived from the six individual donors and were designated as donor-derived samples 1–6. In addition, equal amounts of fecal material from all six donors were pooled, diluted to the same microbial concentration as the donor-derived inocula, divided into three aliquots, and fermented separately to generate three pooled-composite samples, designated as samples 7–9. Thus, each group consisted of nine sequenced fermentation samples, including six donor-derived samples and three pooled-composite samples. All nine samples were included in the 16S rRNA gene sequencing-based analyses. However, the three pooled-composite samples were not considered additional independent donor-derived biological replicates; rather, they were included to provide supplementary information on the pooled microbial community response and overall community characterization. The fecal samples were mixed with normal saline and centrifuged at 200× *g* for 5 min. The supernatant was then collected and centrifuged again at 3000× *g* for 5 min to obtain the bacterial pellet. The bacteria were resuspended and subsequently inoculated into an anaerobic fermentation container for fermentation. Dietary intake was not strictly controlled/recorded, which may contribute to inter-individual variability; this is noted as a limitation.

### 2.2. In Vitro Fermentation of HA Samples

HA samples with average MWs of 6.9, 9.5, 13, 17 and 35 kDa were provided by Shandong Focusfreda Biotechnology Corporation Limited (Shandong, China). The anaerobic fermentation medium contained 8.0 g/L soluble starch, 4.5 g/L yeast extract, 3.0 g/L tryptone, 3.0 g/L peptone, 0.4 g/L bile salts No. 3, 0.8 g/L L-cysteine hydrochloride, 0.5 g/L hemin, 4.5 g/L NaCl, 2.5 g/L KCl, 0.45 g/L MgCl_2_·6H_2_O, 0.2 g/L CaCl_2_·6H_2_O, 0.4 g/L KH_2_PO_4_, 1 mL Tween 80, and 2 mL standard trace element solution, and the pH was adjusted to 6.5 (All reagents used in the experiments, except for hyaluronic acid, were purchased from Sinopharm Group Co., Ltd., Shanghai, China and were of analytical grade. D2O (99.9 atom % D) was obtained from Beijing InnoChem Science & Technology Co., Ltd., Beijing, China). Each HA was added to the medium at a final concentration of 2 g/L [20]. After sterilization, the medium was transferred into anaerobic fermentation vessels, inoculated with the fecal microbiota suspension, and incubated under anaerobic conditions for 48 h. Soluble starch served as a constant background carbohydrate source across the control and all HA-supplemented fermentations; therefore, observed between-group differences reflect the incremental effect attributable to HA supplementation under an identical basal medium. The present design was intended to evaluate the modulation effect of HA supplementation within a standardized fermentation background rather than to assess HA as the sole carbon source.

### 2.3. 16S rRNA Gene Sequencing and Data Analysis

The fermentation broth was collected after 48 h of fermentation and immediately stored at −80 °C. Bacterial DNA was extracted from all fermentation samples in each group, including six donor-derived samples (samples 1–6) and three pooled-composite samples (samples 7–9). Sequencing was performed by BGI Genomics Co., Ltd. (Wuhan, China). After quality filtering, the reads were denoised using the DADA2 pipeline to infer amplicon sequence variants (ASVs) at single-nucleotide resolution [21]. Taxonomic assignment of ASVs was performed using the Ribosomal Database Project (RDP) database [22]. The resulting ASV table was used for downstream analyses of community composition and diversity. Alpha-diversity indices, including ACE, Chao1, Shannon, and Simpson, were calculated from the ASV table to evaluate microbial richness and diversity. Beta-diversity was assessed based on weighted UniFrac distances, and UPGMA clustering analysis was performed to compare similarities and differences in microbial community composition among samples [23]. Differentially abundant taxa were identified using Linear Discriminant Analysis Effect Size (LEfSe) [24], and taxa with higher LDA scores were considered potential microbial biomarkers. Functional prediction of microbial communities was performed using Phylogenetic Investigation of Communities by Reconstruction of Unobserved States 2 (PICRUSt2) [25], and the predicted functional profiles were annotated against the KEGG database to obtain pathway abundance at the community level [26]. Microbial correlation network analysis was conducted using SparCC based on species abundance data [27], and Spearman correlation analysis was performed to evaluate associations between dominant bacterial genera and SCFA concentrations. Unless otherwise specified, 16S rRNA gene sequencing-based analyses included nine sequenced fermentation samples per group, consisting of six donor-derived samples from independent donors and three separately fermented pooled-composite samples. The pooled-composite samples were included in the sequencing dataset but were not considered additional independent donor-derived biological replicates.

### 2.4. SCFAs Analysis

We determined the concentration of SCFAs using UPLC. We employed an Inertsustain Aq-C18 column (150 mm × 2.1 mm, 1.9 μm) with a flow rate of 0.5 mL/min. We set the UV detector to 210 nm, maintained the column temperature at 40 °C, and used an injection volume of 10 μL. We prepared the mobile phase as an acetonitrile-NaH_2_PO_4_ system, consisting of Solution A (20 mmol/L NaH_2_PO_4_, pH adjusted to 2.2 with H_3_PO_4_) and Solution B (acetonitrile). Prior to use, we filtered both solutions through 0.22 μm membranes and performed ultrasonic degassing for 30 min. For sample preparation, we mixed 2 mL of fermentation broth with 0.2 mL of 2 mol/L H_2_SO_4_. We then vortexed the mixture vigorously for 1 min and incubated it at room temperature for 30 min. Following incubation, we centrifuged the sample at 12,000× *g* for 20 min at 4 °C. We collected the supernatant, filtered it through a 0.22 μm hydrophilic membrane, and transferred the filtrate to a UPLC vial for analysis. We used the following elution program: from 0 to 10 min, we linearly decreased Solution A from 95% to 60%; we then maintained this ratio from 10 to 12 min to complete the run. SCFA concentrations were determined once for each fermentation sample. No technical replicate measurements were performed for SCFA quantification.

### 2.5. Statistical Analysis

Statistical analyses were performed using GraphPad Prism (version 8.0.1). For comparisons among three or more groups, the normality of data distribution was first assessed using the Shapiro–Wilk test. Data with normal distribution were analyzed by one-way ANOVA, followed by Tukey’s post hoc test for pairwise comparisons. Data not conforming to normal distribution were analyzed by the Kruskal–Wallis test, followed by Dunn’s post hoc test for pairwise comparisons. The False Discovery Rate was controlled for multiple comparisons using the two-stage step-up method of Benjamini, Krieger, and Yekutieli. All data are presented as mean ± standard deviation (SD). A *p*-value of <0.05 was considered statistically significant.

## 3. Results

### 3.1. Effects of HAs on the Structure of Gut Microbiota

To investigate the effects of HA on gut microbiota, we processed the sequencing data using the DADA2 algorithm to generate ASVs clustered at 100% similarity. Species richness curves, rarefaction curves, and rank abundance curves confirmed adequate sampling depth, reliable sequencing data, and representative species diversity. At the phylum level, Bacillota, Pseudomonadota and Bacteroidota dominated the microbial community. At the genus level, the abundance of *Bifidobacterium* was significantly higher in the HA groups than in the control group (*p* < 0.05), showing a positive correlation with HA MW. Unless otherwise specified, 16S rRNA gene sequencing-based analyses included nine sequenced fermentation samples per group, consisting of six donor-derived samples from independent donors and three separately fermented pooled-composite samples. The pooled-composite samples were included in the sequencing dataset to provide supplementary information on the pooled microbial community response and overall community characterization, but they should not be interpreted as additional independent donor-derived biological replicates.

#### 3.1.1. Alpha and Beta Diversity Changes

**Figure 1:**
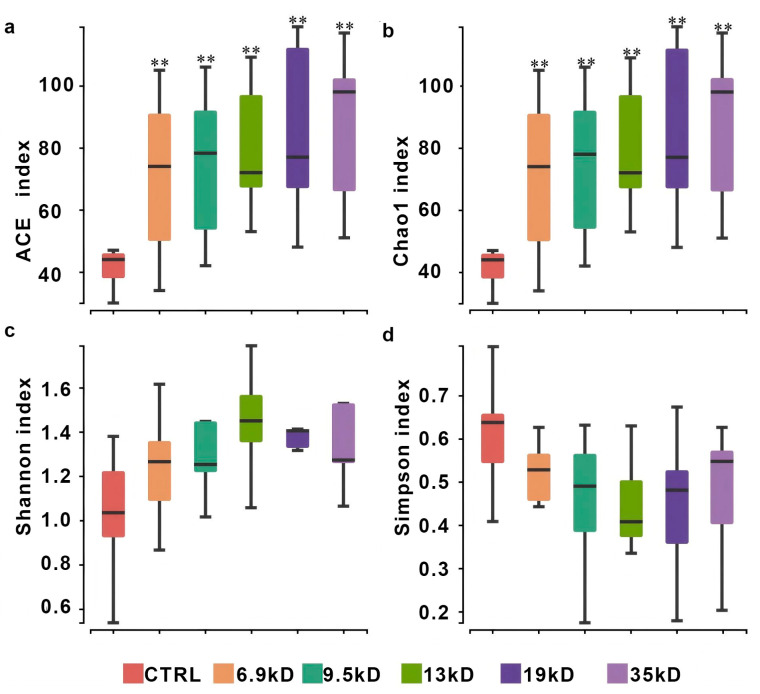
Alpha diversity of gut microbiota communities across the experimental groups. (**a**) ACE index; (**b**) Chao1 index; (**c**) Shannon index; (**d**) Simpson index. The *x*-axis shows the experimental groups: control group (CTRL), HA-6.9 kDa, HA-9.5 kDa, HA-13 kDa, HA-17 kDa, and HA-35 kDa. Each group included nine sequenced fermentation samples, consisting of six donor-derived samples from independent donors (samples 1–6) and three separately fermented pooled-composite samples (samples 7–9). ** *p* < 0.01 compared with CTRL.

**Figure 2:**
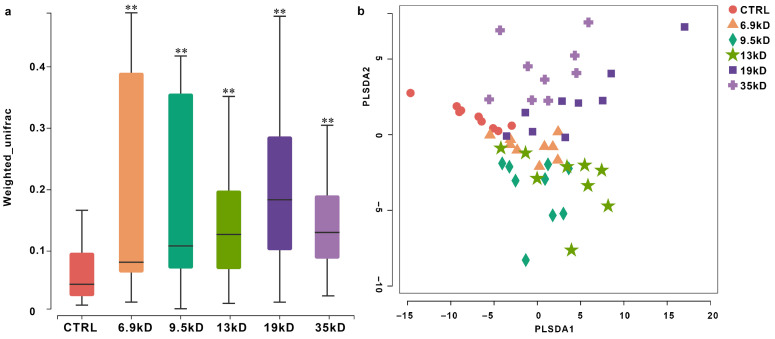
Beta diversity analysis of gut microbiota communities across different groups. (**a**) Box plot based on weighted UniFrac distances, showing the distribution of distances and inter-group variation. ** *p* < 0.01 compared with the CTRL. (**b**) Partial Least Squares Discriminant Analysis (PLS-DA) plot illustrating the separation of microbial community structures among groups. Each group included nine sequenced fermentation samples, consisting of six donor-derived samples from independent donors (samples 1–6) and three separately fermented pooled-composite samples (samples 7–9).

To examine whether HA MW is associated with differences in gut microbial community structure, we analyzed alpha-diversity and beta-diversity across a fine gradient of HA polymers (6.9–35 kDa). ACE and Chao1 were significantly elevated in all HA-supplemented groups compared to the control, suggesting that HA supplementation was associated with higher richness under the present in vitro conditions. (Figure 1) We further observed that the degree of increase in diversity was not uniform but followed an MW-dependent trend, with lower-MW HA groups (e.g., 6.9, 9.5 kDa) consistently exhibiting higher values than higher-MW groups (e.g., 17, 35 kDa), suggesting that shorter HA chains were associated with relatively higher alpha-diversity indices under the present experimental conditions. Beta-diversity differences were primarily evaluated using weighted UniFrac distances with PERMANOVA. PLS-DA was applied as a complementary supervised visualization approach to illustrate group separation patterns. (Figure 2a,b). Ordination suggested MW-related separation patterns, indicating that different HA MWs were associated with shifts in community structure. Specifically, samples from lower- and higher-MW HA groups showed partially separated clustering patterns, suggesting differences in taxonomic composition and potential ecological interaction patterns associated with polymer chain length. Permutational multivariate analysis of variance (PERMANOVA) confirmed significant differences among groups (*p* < 0.05).

#### 3.1.2. Effects of HAs on the Structure of the Gut Microbiota: MW-Specific Shifts

**Figure 3:**
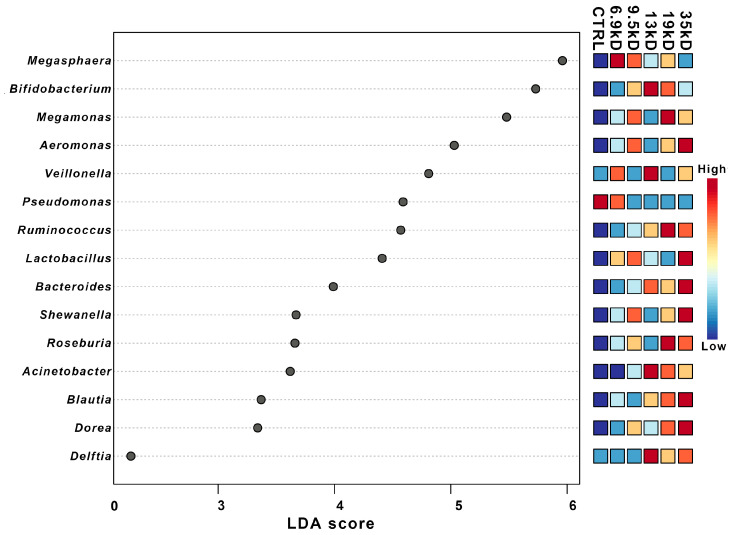
Difference analysis of gut microbiota composition LDA value distribution histogram between the HA groups and the CTRL. CTRL is the blank control group, and HA (6.9–35 kD) is the HA group. LDA score greater than 2, significant *p* < 0.05. Taxonomic enrichment does not directly imply metabolic activity but reflects shifts in microbial community composition. Points labeled “High” and “Low” indicate the relative LDA scores of certain taxa across groups.

To identify the specific bacterial taxa responding to HA of different sizes, we performed Linear Discriminant Analysis Effect Size analysis. The results identified several bacterial genera with differential relative abundance in the HA-treated groups compared with the control group and suggested an MW-associated enrichment pattern (Figure 3). Specifically, fermentation with the lowest-molecular-weight HA (6.9 kDa) was characterized by the enrichment of genera such as *Megasphaera*, *Veillonella*, and *Pseudomonas*, which are commonly reported to participate in the utilization of fermentation intermediates, including lactate and other organic acids.

In contrast, fermentation with the 17 kDa and 35 kDa consistently enriched genera including *Bifidobacterium*, *Megamonas*, *Ruminococcus*, and *Lactobacillus*, taxa that are typically associated with the degradation and utilization of complex carbohydrates. These differences in taxonomic composition suggest that HA MW may be associated with shifts in gut microbial community structure, with enrichment of taxa that have been reported in previous studies to participate in different carbohydrate-related ecological niches. Supporting this observation, correlation analysis demonstrated that the relative abundances of *Ruminococcus* and *Blautia* were positively correlated with increasing HA MW, whereas *Megamonas* exhibited a negative correlation. Together, these opposing trends indicate that variation in HA chain length was associated with differences in the relative abundance of taxa previously linked to polysaccharide degradation or secondary fermentation-related processes. Such molecular-weight-dependent shifts in community composition may be related to the distinct metabolic outcomes observed among different HA treatments, as reflected in the subsequent SCFA production profiles.

#### 3.1.3. Predicted Functional Profiling Based on 16S rRNA Gene Data

**Figure 4:**
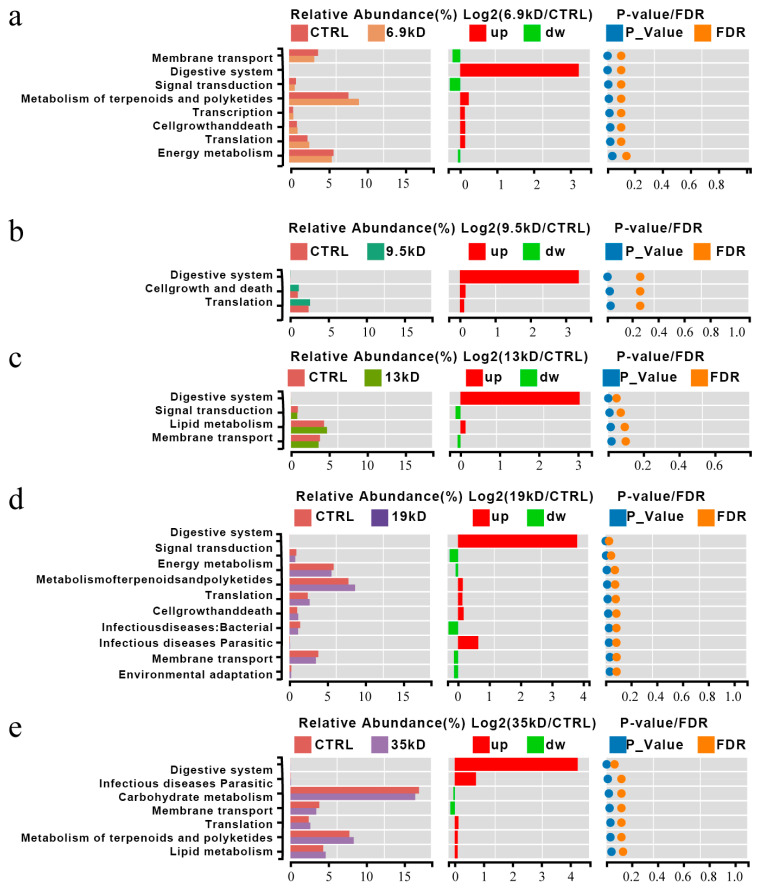
Functional difference predictive analysis in gut microbiota between the HA group and the CTRL. based on KEGG level 2 pathway annotation. (KEGG pathways were inferred from 16S rRNA gene data and should be interpreted only as predicted functional potential.). On the left is the relative abundance histogram of each group; in the middle is the log2 value of the mean relative abundance ratio of the same pathway in the two groups; on the right is the *p*-value obtained by the Wilcox test. (**a**) CTRL Vs HA-6.9 kDa; (**b**) CTRL Vs 9.5 kDa; (**c**) CTRL Vs 13 kDa; (**d**) CTRL Vs 19 kDa; (**e**) CTRL Vs 35 kDa. The left panel shows a histogram of the relative abundance for each group; the middle panel shows the log2 value of the ratio of mean relative abundance of the same pathway between the two groups; and the right panel shows the *p*-values obtained by the Wilcox test.

Functional profiles were inferred using 16S-based prediction and should be interpreted as predicted functional potential rather than directly measured pathway activity. Therefore, these analyses cannot substitute for metagenomic, metatranscriptomic, or metabolomic validation of actual pathway function. To explore the potential functional consequences of MW-dependent microbial shifts, we annotated predicted functions using KEGG pathways. While HA supplementation broadly increased the predicted abundance of fundamental metabolic pathways (e.g., digestive system, translation) across all groups, a molecular-weight-dependent divergence in predicted functional profiles was observed (Figure 4).

In communities fermenting the 17 and 35 kDa HA groups, we detected a relative decrease in predicted membrane transport pathways accompanied by an increase in pathways annotated to terpenoid and polyketide metabolism. Although these predictions do not directly reflect actual metabolic activity, this pattern may be associated with differences in gene content among taxa enriched under higher-MW conditions, such as *Bifidobacterium* and *Ruminococcus*. These genera have been reported to be associated with complex carbohydrate-related niches, and the observed predicted functional differences may reflect variation in community composition rather than directly demonstrating adaptation to larger polymeric substrates.

In contrast, communities fermenting the 6.9 and 9.5 kDa HA groups exhibited relatively higher predicted representation of central carbohydrate metabolism pathways, including glycolysis/gluconeogenesis and pyruvate metabolism. Given that these pathways are broadly involved in carbohydrate metabolism and fermentation-associated processes, their predicted enrichment is consistent with the elevated SCFA levels measured in these groups, but does not demonstrate increased metabolic flux. However, as these results are based on predictive inference from 16S data, they should not be interpreted as direct evidence of increased metabolic flux toward SCFA biosynthesis.

Thus, the KEGG-based prediction analysis suggests that HA molecular weight may be associated with distinct functional configurations at the community level. Higher-MW HAs appear to be linked with predicted functional categories related to complex substrate processing, whereas lower-MW HAs are associated with greater predicted central metabolic potential. Because the present system did not isolate HA as the sole substrate, these predicted functional distinctions should be interpreted in the context of HA-associated modulation under a shared basal medium. They therefore provide a hypothesis-generating framework that may help explain differences in SCFA production patterns, pending validation by metagenomic or metabolomic approaches.

#### 3.1.4. Species-Level Interconnections

**Figure 5:**
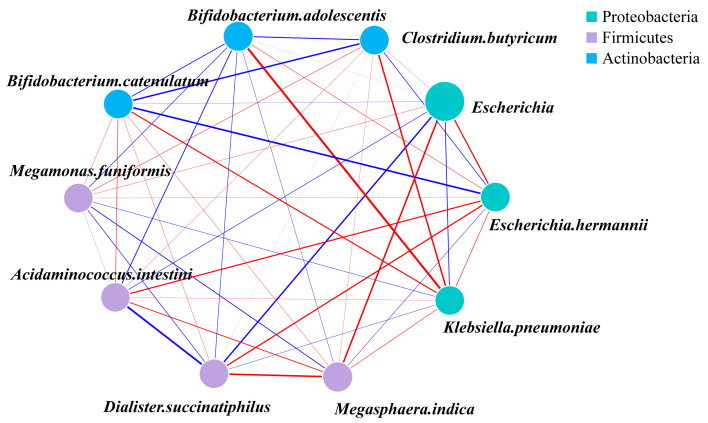
Species-level gut microbiota correlation networks. red edges indicate positive correlations, blue edges indicate negative correlations.

To explore whether HA MW was associated with changes in species-level co-occurrence patterns, we constructed a correlation network (Figure 5). Correlation coefficients were calculated using ASV abundance data from the nine sequenced fermentation samples in each group, including six donor-derived samples and three pooled-composite samples. The pooled-composite samples were included to support overall co-occurrence pattern characterization, but they should not be interpreted as additional independent donor-derived biological replicates. The network suggested that species-level co-occurrence patterns differed among HA treatment groups. In the context of lower-MW HA fermentation, we observed a tightly linked module featuring *Clostridium butyricum* and *Megamonas funiformis*. Their strong positive correlation may indicate a potential ecological association relevant to butyrate-related community metabolism, but does not by itself demonstrate metabolic syntrophy or causality. Conversely, in the higher-MW HA groups, a significant negative correlation was observed between two abundant *Bifidobacterium* species (*Bifidobacterium adolescentis* and *Bifidobacterium catenulatum*), which may reflect divergent co-occurrence patterns rather than direct evidence of competition.

This pattern may reflect differences in ecological niches among taxa, although direct evidence for competition or resource partitioning was not obtained in the present study. Notably, *Bifidobacterium adolescentis* also showed a negative correlation with *Acidaminococcus intestini*, a pattern distinct from its relative, further suggesting that HA MW may be associated with different co-occurrence patterns among taxa occupying related ecological niches. Collectively, these interaction patterns suggest that HA MW may be associated not only with changes in the abundance of individual species but also with broader differences in co-occurrence patterns within the microbial community. These network-level differences provide an ecological context for interpreting the distinct, MW-dependent community assemblies and their associated metabolic outputs, but they should not be interpreted as direct evidence of cooperation, competition, or mechanism.

### 3.2. Effects of HA on the Production of SCFAs in In Vitro Fermentation

**Figure 6:**
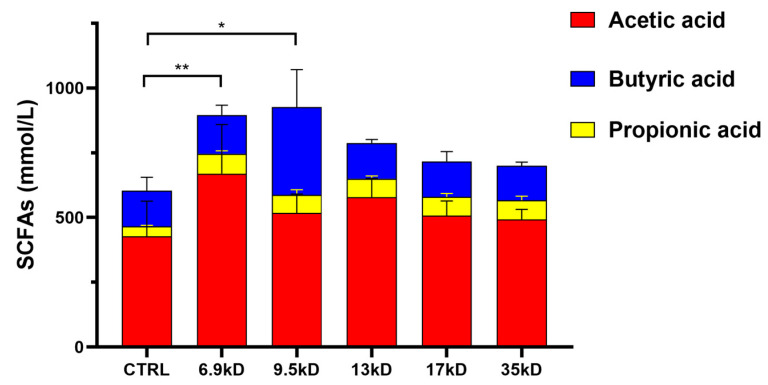
Concentrations of short-chain fatty acids (acetate, propionate, and butyrate) in the fermentation broth after 48 h of in vitro anaerobic fermentation. Each group included nine fermentation bottles, consisting of six donor-derived fermentation samples and three separately fermented pooled-composite samples. SCFA concentrations were determined once for each fermentation bottle. * *p* < 0.05, ** *p* < 0.01 compared with the CTRL.

It should be emphasized that soluble starch likely served as the dominant carbon source in the system; therefore, the observed SCFA differences represent modulation effects of HA supplementation rather than direct fermentation of HA as a sole substrate. To quantify the MW-dependent differences in fermentation-associated output under these conditions, SCFA production was determined after 48 h of in vitro incubation. UPLC analysis showed that HA supplementation was associated with changes in SCFA profiles compared with the control, with an MW-associated pattern (Figure 6). SCFA concentrations are presented as mean ± standard deviation (SD) based on nine fermentation bottles per group, including six donor-derived fermentation samples and three separately fermented pooled-composite samples. The 6.9 and 9.5 kDa samples were associated with significantly higher total SCFA concentrations, particularly butyrate, than 13, 17 and 35 kDa samples, indicating stronger SCFA-associated output within this MW range under the present fermentation background.

One possible explanation for the higher SCFA levels observed in the lower-MW HA groups is that HA MW may influence its interaction with microbial communities within the shared fermentation background. However, specific HA degradation kinetics, residual substrate levels, and enzymatic activities were not directly examined in the present study. Therefore, these interpretations should be considered plausible explanations rather than experimentally confirmed mechanisms.

The elevated butyrate levels in the lower-MW groups were accompanied by coordinated changes in multiple bacterial genera, suggesting that SCFA formation may reflect integrated community-level metabolic activity rather than the dominance of a single taxon. From a functional food perspective, these findings suggest that HA molecular weight may be a relevant formulation parameter associated with microbiota-modulating and SCFA-related outcomes in vitro. However, the present findings should be interpreted as MW-dependent modulation under a starch-containing fermentation background rather than proof of HA as the primary carbon source or direct evidence of HA-specific fermentation.

### 3.3. Correlations Between Dominant Bacterial Genera and SCFA Production

**Figure 7:**
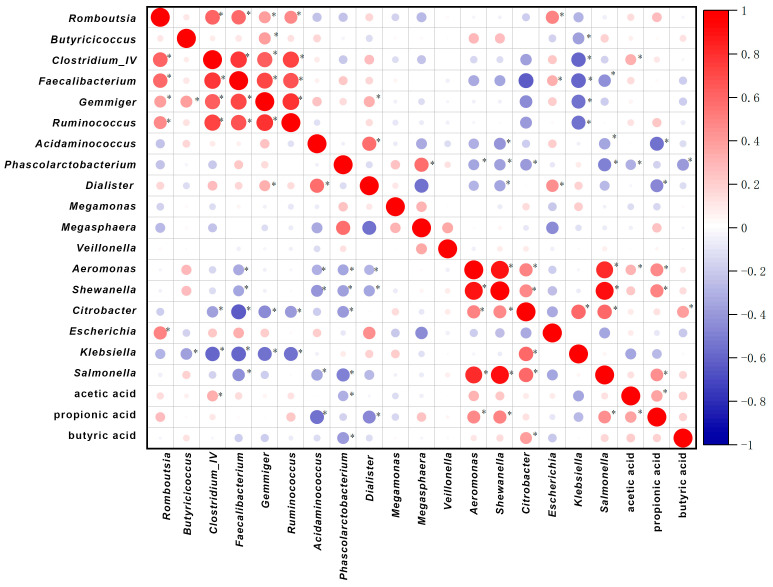
Spearman correlation matrix showing significant associations between bacterial genera and SCFA concentrations. * *p* < 0.05.

To further explore the relationships between microbial community composition and SCFA production, Spearman correlation analysis was performed between the relative abundances of dominant bacterial genera and the concentrations of individual SCFAs (Figure 7). Acetate and propionate exhibited significant positive correlations with multiple commensal taxa, including *Clostridium_IV*, indicating that their production was broadly associated with overall microbial fermentation activity within the system. These widespread correlations suggest that acetate and propionate accumulation reflect general metabolic activity rather than the contribution of a limited number of specific taxa.

In contrast, butyrate displayed significant correlations with only a small subset of bacterial genera. Notably, *Clostridium_IV* and *Veillonella* were positively correlated with butyrate concentrations, whereas *Phascolarctobacterium* and *Dialister* showed significant negative correlations. Meanwhile, several well-recognized butyrate-associated genera, such as *Faecalibacterium* and *Ruminococcus*, did not exhibit significant linear correlations with butyrate levels in this in vitro fermentation system. This observation suggests that the abundance of individual butyrate-associated taxa alone is insufficient to explain variations in butyrate production under the experimental conditions used in this study.

Collectively, the correlation analysis indicates that SCFA production, particularly butyrate formation, cannot be attributed to the abundance of any single bacterial genus. Although a limited number of taxa, such as *Clostridium_IV* and *Veillonella*, showed significant positive correlations with butyrate levels, several genera commonly regarded as butyrate-associated did not display significant relationships. At the same time, the presence of both positive and negative correlations among different taxa suggests that butyrate accumulation depends on the balance between multiple microbial populations with distinct metabolic roles. In contrast, acetate and propionate were correlated with a wider range of dominant genera, indicating a closer association with overall fermentative activity. Together, these patterns support the interpretation that SCFA profiles reflect integrated, community-level metabolic processes rather than simple one-to-one relationships between individual taxa and specific SCFAs. However, the present correlation analysis does not establish causality or direct metabolic mechanism.

## 4. Discussion

### 4.1. MW-Dependent Microbial and SCFA Shifts Associated with LMW HA Under a Starch-Containing Fermentation Background

The present study provides a microbiota-centered view of how fine-scale variation in the molecular weight of low-molecular-weight hyaluronic acid (LMW HA) is associated with differential fermentation outcomes in vitro. Within the tested range (6.9–35 kDa), lower-MW HA samples, particularly 6.9 and 9.5 kDa, were associated with higher total SCFA concentrations and greater butyrate accumulation than higher-MW samples under the same fermentation background. These findings are broadly consistent with previous reports showing that HA can be utilized by the gut microbiota and that HA molecular weight influences microbial community structure, HA degradation, and fermentation-derived metabolite output [8,9,18,19].

Importantly, our study extends previous broad-range molecular-weight comparisons by focusing on a fine gradient within the LMW window. This design suggests that even relatively small differences in HA chain length may be linked to distinct microbial configurations and SCFA output patterns. Similar MW-dependent differences in gut microbiota fermentation characteristics have also been reported for other dietary polysaccharides, supporting the broader concept that polymer size can influence microbial fermentation behavior [28]. A central limitation of the present study, however, is that soluble starch was included in the basal medium and likely acted as the dominant fermentable carbohydrate. Consequently, the current design cannot conclusively demonstrate HA-specific fermentation or establish a direct causal relationship between HA degradation and SCFA formation. Instead, the observed differences among HA groups are more appropriately interpreted as MW-dependent modulation effects of HA supplementation under a starch-containing fermentation background. Within this framework, one possible explanation is that HA MW may influence how HA interacts with the microbial community under the shared fermentation background. However, because HA degradation kinetics, residual HA consumption, and enzyme activities were not directly measured, the present study cannot determine whether the higher SCFA levels in the lower-MW groups resulted from greater HA accessibility or more efficient microbial processing [9,18,19].

Our taxonomic and correlation analyses further suggest that the metabolic consequences of HA supplementation should be interpreted at the community level rather than being attributed to a single bacterial genus. Although lower-MW HA was associated with enrichment of taxa such as *Megasphaera* and *Veillonella*, and higher-MW HA was associated with enrichment of *Bifidobacterium*, *Megamonas*, *Ruminococcus*, and *Lactobacillus*, these compositional shifts do not by themselves demonstrate specific metabolic activity. In the gut ecosystem, SCFA production, especially butyrate formation, often depends on cross-feeding interactions among primary degraders, intermediate metabolite producers, and secondary fermenters rather than on one taxon acting alone [29,30]. However, such interactions were not directly tested in the present study. This may explain why butyrate concentrations in the present study were not fully accounted for by the abundance of any single classical butyrate-associated genus.

The biological relevance of the elevated butyrate observed in the lower-MW HA groups deserves particular emphasis. Butyrate is a major microbiota-derived metabolite involved in epithelial energy metabolism, mucosal homeostasis, intestinal barrier maintenance, and immune regulation [31]. Consistent with this concept, a recent study published in iMeta demonstrated that butyrate can improve intestinal barrier integrity through suppression of oxidative stress-mediated Notch signaling, further supporting the functional importance of butyrate beyond its role as a fermentation end product [32]. From this perspective, the higher butyrate levels associated with lower-MW HA may provide a rationale for future host-relevant studies, although the present in vitro system cannot determine whether these SCFA differences translate into physiological benefits.

Another important issue concerns the functional prediction results. In this study, KEGG-based analyses were inferred from 16S rRNA gene data using PICRUSt2 and therefore should be interpreted as predicted functional potential rather than directly measured pathway activity [25]. Such analyses are useful for generating hypotheses, but they cannot substitute for metagenomic, metatranscriptomic, or metabolomic validation of actual pathway function. Recent benchmarking work has shown that 16S rRNA gene-based metagenome prediction has limited sensitivity for capturing subtle functional differences and that the accuracy of metagenome prediction tools can vary across sample types and functional categories; such results should therefore be interpreted cautiously [33,34]. Accordingly, the predicted enrichment of central carbohydrate metabolism in the lower-MW groups should be viewed as hypothesis-generating rather than as direct evidence of increased metabolic flux. Validation by metagenomics, metatranscriptomics, targeted metabolomics, or direct enzymatic assays will be necessary to clarify the molecular mechanisms underlying the MW-dependent differences observed here.

### 4.2. Broader Implications, Study Limitations, and Future Directions

More broadly, our findings fit within the growing concept that food-derived bioactive materials can influence host physiology through coordinated modulation of gut microbiota and microbial metabolites [35]. Although that study did not involve HA, it illustrates the broader relevance of microbiota–metabolite interactions in evaluating functional food materials. Taken together, the present study suggests that HA molecular weight may be an important formulation parameter influencing microbiota-associated fermentation responses under in vitro conditions. Several limitations of the present study should also be acknowledged. First, the starch-containing basal medium prevents definitive attribution of SCFA production to HA as a sole substrate. Second, the number of donor-derived biological replicates was limited to six, which constrains statistical power, may not fully capture inter-individual microbiota variability, and limits the generalizability of the findings. In addition, three samples in each group were derived from pooled inocula rather than independent donors. Although these pooled-composite samples were included in the sequencing-based analyses and were useful for complementary community characterization, they do not provide additional donor-level biological variability and should therefore be interpreted with caution. Third, environmental parameters of fermentation, such as pH and redox potential, were not systematically monitored. Fourth, reliance on 16S-based functional prediction does not permit direct inference of pathway activity. Future studies should therefore combine carbohydrate-minimized or starch-free fermentation systems with direct HA degradation assays, metagenomics, targeted metabolomics, and host-relevant validation models to clarify whether and how HA MW contributes to fermentation-associated microbial and metabolic outcomes.

## 5. Conclusions

In a starch-containing in vitro fecal fermentation model, supplementation with fine-gradient LMW hyaluronic acid (6.9–35 kDa) was associated with MW-dependent shifts in microbial community structure and fermentation-derived SCFA profiles. The 6.9 and 9.5 kDa HA groups consistently resulted in higher total SCFA concentrations, particularly butyrate, than the 13, 17 and 35 kDa HA groups. These differences coincided with coordinated changes across multiple taxa linked to carbohydrate utilization and secondary fermentation, supporting a community-level interpretation of butyrate accumulation rather than a single-taxon effect. Together, our findings should be interpreted as evidence of MW-dependent modulation under a starch-containing in vitro fermentation background rather than direct proof of HA-specific fermentation. In addition, the 16S-derived functional results should be regarded as predicted potential only and will require validation by metagenomic or metabolomic approaches before functional conclusions can be established. Because substrate utilization, HA degradation kinetics, and enzyme activities were not directly measured, the present study cannot establish a causal mechanism linking HA MW to microbial metabolism or SCFA production.

## Data Availability

The datasets generated and analyzed during the current study are available from the corresponding author on reasonable request.

## Abbreviations

The following abbreviations are used in this manuscript:

UPLC	Ultra Performance Liquid Chromatography
SCFA	Short-chain fatty acids
HMW	High-molecular-weight
LMW	Low-molecular-weight
HA	Hyaluronic Acid
MW	Molecular Weight
ASV	Amplicon Sequence Variant
PLS-DA	Partial Least Squares Discriminant Analysis
PERMANOVA	Permutational Multivariate Analysis of Variance
KEGG	Kyoto Encyclopedia of Genes and Genomes
LEfSe	Linear Discriminant Analysis Effect Size
ANOVA	Analysis of Variance
RDP	Ribosomal Database Project
ISAPP	International Scientific Association for Probiotics and Prebiotics
CTRL	control group
PICRUSt2	Phylogenetic Investigation of Communities by Reconstruction of Unobserved States 2
